# Neuropsychological tests at the Italian Centers for Cognitive Disorders and Dementias: results from a survey on 450 specialized services

**DOI:** 10.1007/s40520-024-02869-6

**Published:** 2024-12-20

**Authors:** Roberta Vaccaro, Patrizia Lorenzini, Francesco Giaquinto, Fabio Matascioli, Giulia Carnevale, Francesco Sciancalepore, Marina Gasparini, Emanuela Salvi, Massimo Corbo, Nicoletta Locuratolo, Nicola Vanacore, Ilaria Bacigalupo, The Permanent Table of the National Dementia Plan Study Group, Gennarina Arabia, Alessandro Amorosi, Ilaria Bacigalupo, Anna Maria Bargagli, Luisa Bartorelli, Cristina Basso, Manuela Berardinelli, Maria Pompea Bernardi, Caterina Bianchi, Lorenzo Blandi, Federica Boschi, Amalia Cecilia Bruni, Alessandra Caci, Paolo Caffarra, Marco Canevelli, Andrea Capasso, Susanna Cipollari, Mariapia Cozzari, Alfonso Di Costanzo, Teresa Di Fiandra, Annalisa Di Palma, Andrea Fabbo, Federica Francescone, Carlo Gabelli, Sabina Gainotti, Francesca Galeotti, Giuseppe Gambina, Marina Gasparini, Maria Assunta Giannini, Micaela Gilli, Marcello Giordano, Annarita Greco, Antonio Guaita, Fabio Izzicupo, Fiammetta Landoni, Elisa Lidonnici, Nicoletta Locuratolo, Giancarlo Logroscino, Alessandra Lombardi, Gilda Losito, Francesca Lubian, Maria Cristina Lupinetti, Sara Madrigali, Camillo Marra, Filippo Masera, Massimiliano Massaia, Antonio Mastromattei, Antonio Matera, Manlio Matera, Francesco Mazzoleni, Carla Melani, Serena Meloni, Elena Memeo, Marco Musso, Antonella Notarelli, Marco Onofrj, Ernesto Palummeri, Valeria Panetta, Carlo Petrini, Tommaso Piccoli, Alessandro Pirani, Stefano Piras, Gabriella Porro, Mario Possenti, Elena Rendina, Antonino Riolo, Luciana Riva, Emanuela Salvi, Sara Santini, Silvia Scalmana, Nando Scarpelli, Piero Secreto, Monica Seganfreddo, Stefano Sensi, Carla Severino, Patrizia Spadin, Patrizia Spallino, Anna Laura Spinelli, Andrea Stracciari, Marco Trabucchi, Nicola Vanacore, Antonio Zaccardi, The CCDDs Study Group, Egidio Accardo, Omar Ahmad, Domenico Ajena, Giovanni Alba, Alberto Albanese, Andrea Albergati, Maria Alessandria, Pasquale Alfieri, Mario Alimenti, Angelo Aliprandi, Roberto Altavilla, Salvatore Amarù, Immacolata Ambrosino, Felice Amideo, Stefania Ammendola, Francesco Amoruso, Candida Andreati, Vincenzo Andreone, Rossano Angeloni, Francesco Annunziata, Sara Antenucci, Ildebrando Appollonio, Gennarina Arabia, Luciano Arcudi, Marianna Ardillo, Maria Carmela Gabriella Arena, Andrea Arighi, Gennaro Arpino, Anna Bagalà, Antonio Baiano, Antonio Balestrino, Mario Barbagallo, Marianna Barbuto, Cesare Bargnani, Paolo Barone, Antonella Bartoli, Claudia Bauco, Giuseppe Bellelli, Marco Antonio Bellini, Aldo Bellora, Giuseppe Benati, Sandro Beretta, Lucia Bergamini, Eleonora Bergonzini, Valentina Bessi, Angelo Bianchetti, Erika Bisio, Roberta Boiardi, Elisabetta Bollani, Laura Bologna, Francesco Bolzetta, Stefano Boni, Tiziano Borgogni, Gabriella Bottini, Ida Bottone, Angela Bove, Roberto Bruno Bossio, Giuseppe Bruno, Patrizia Bruno, Carmela Bucca, Manuela Buganza, Graziano Buzzi, Paolo Buzzi, Gabriella Cacchio’, Arturo Cafarelli, Viviana Cafazzo, Marcella Caggiula, Annachiara Cagnin, Gianluigi Calabrese, Giusi Alessandro Calabrese, Maria Calandra, Veronica Caleri, Donatella Calvani, Massimo Camerlingo, Roberto Cantello, Andrea Capasso, Sabina Capellari, Giovanni Capobianco, Maria Carmela Capoluongo, Rossana Cappelletti, Claudio Capra, Natalia Caravona, Carlo Maria Stucchi, Maria Alessandra Carluccio, Severina Carteri, Anna Casanova, Francescosaverio Caserta, Paolo Caso, Gaetana Cassaniti, Emanuele Cassetta, Silvia Casson, Vincenzo Castiello, Tatiana Cattaruzza, Anna Ceccon, Moira Ceci, Sabatino Cella, Silvia Cenciarelli, Bruno Censori, Giuliano Cerqua, Paolo Cerrone, Pasquale Cervera, Silvia Chemotti, Annalisa Chiari, Roberta Chiloiro, Luisa Cirilli, Raffaella Clerici, Alessandra Coin, Gianfranco Colacino, Francesco Paolo Colacioppo, Rosanna Colao, Antonio Colin, Brigida Coluccia, Giancarlo Maria Conti, Filomena Coppola, Francesca Coppola, Massimo Corbo, Antonello Cossu, Alfredo Costa, Gabriella Costa, Manuela Costa, Maria Sofia Cotelli, Salvatore Cottone, Maria Immacolata Cozzolino, Andrea Crucitti, Eduardo Cumbo, Antonio Currà, Carlo Dallocchio, Ferdinando D’amico, Anna D’Amore, Stefano De Carolis, Maurizio De Donato, Paola De Feo, Franz De La Pierre, Maria De Laurentiis, Ida De Lauretis, Gian Placido De Luca, Alessandro De Palma, Laura De Togni, Antonio Demontis, Dora D’Epiro, Giovambattista Desideri, Miranda Desiderio, Marco Di Donato, Gabriella Di Emidio, Raffaella Di Giacopo, Vincenzo Di Lazzaro, Rita Di Leo, Salvatore Di Marco, Gaetano Di Quarto, Babette Dijk, Natasa Dikova, Maria Stefania Dioguardi, Federica Dominici, Michele Dotta, Carla Dotti, Domenica Esposito, Sabrina Esposito, Zaira Esposito, Evaristo Ettorre, Andrea Fabbo, Giovanna Faccenda, Angelamaria Falanga, Michela Falorni, Fraia Falvo, Agostina Fappani, Elisabetta Ismilde Mariagiovanna Farina, Sara Fascendini, Francesco Fattapposta, Grazia Daniela Femminella, Salvatore Ferrara, Patrizia Ferrari, Alessandra Ferraris, Franco Ferraro, Raffaele Ferri, Salvatore Ferrigno, Francesco Filastro, Massimo Filippi, Antonio Finelli, Chiara Finelli, Maria Rita Fiori, Francesco Fiorillo, Gianluca Floris, Anna Fontanella, Luigi Forgione, Andrea Foti, Francesca Fulvia Foti, Fabio Frediani, Giovanni Frontera, Maria Luigia Fulgido, Cira Fundarò, Carmine Fuschillo, Luciano Gabbani, Carlo Gabelli, Franco Galati, Renato Galli, Angelo Gallo, Livia Gallo, Maurizio Gallucci, Gabriella Galluccio, Pietro Gareri, Lorenzo Gasperi, Giovanni Gelmini, Michele Gennuso, Carmela Gerace, Daria Ghersetti, Federica Giambattistelli, Valter Giantin, Bernardo Giordano, Maurizio Giorelli, Agata Giorgianni, Franco Giubilei, Laura Godi, Luciano Gorelli, Daniela Gragnaniello, Serena Granziera, Giuseppe Greco, Rodolfo Grella, Michele Grieco, Luigi Grimaldi, Maria Guarino, Chiara Guarnerio, Giovanni Guidi, Leonello Guidi, Lucia Iallonardo, Alessandro Iavarone, Tiziana Ingegni, Pasqualina Insardà, Claudio Ivaldi, Fabio Izzicupo, Carmelo Roberto Labate, Roberto Lacava, Francesco Lalli, Anna Maria Lammardo, Paolo Massimo Laurienzo, Alessandro Leonardi, Maria Rosa Leotta, Rosario Leuzzi, Simona Linarello, Pasqualino Litterio, Daniele Lo Coco, Mario Rosario Lo Storto, Chiara Logi, Francesco Ottavio Logullo, Alessandra Lombardi, Fortunato Lombardi, Antonio Lorido, Francesco Antonio Losavio, Francesca Lubian, Antonina Luca, Livia Ludovico, Maria Lunardelli, Mariarosaria Lupo, Simona Luzzi, Maurizio Maddestra, Gennaro Maio, Mariangela Maiotti, Anna Maria Malagnino, Giovanni Mancini, Angela Manica, Michele Maniscalco, Barbara Manni, Antonio Manucra, Laura Manzoni, Marco Marabotto, Giuseppe Marchesiello, Michela Marcon, Alessandra Marcone, Roberto Marconi, Alessandro Margiotta, Angela Marianantoni, Donatella Mariani, Gemma Marino, Saverio Marino, Vito Marinoni, Angela Marra, Camillo Marra, Maria Marrari, Mabel Martelli, Alessandro Marti, Alessandro Martorana, Martina Marvardi, Saverio Mascolo, Mario Massimiliano Massaia, Lucio Massimo Lenzi, Vita Maria Alba Mastronuzzi, Maria Letizia Mazzi, Andrea Mazzone, Rossella Mecacci, Patrizia Mecocci, Deidania Medici, Daniele Mei, Gian Giuseppe Melandri, Maurizio Melis, Francesca Meneghello, Vanda Menon, Carmen Menza, Paola Merlo, Graziella Milan, Antonio Milia, Calogero Claudio Millia, Sergio Minervini, Carolina Anna Mobilia, Massimo Moleri, Elena Molteni, Giovanni Moniello, Stefano Montanari, Maria Teresa Mormile, Giuseppe Moro, Gianluca Moscato, Enrico Mossello, Angela Domenica Mundo, Giuseppe Mura, Fabio Musca, Anna Maria Musso, Anna Nardelli, Francesca Neviani, Viviana Nicosia, Vincenzo Nociti, Alessio Novelli, Francesco Nuccetelli, Marco Onofrj, Lorenza Orefice, Daniele Orsucci, Alfonso Pace, Cristina Paci, Roberta Padoan, Alessandro Padovani, Lorenzo Palleschi, Maria Teresa Palmisani, Marco Palmucci, Pasquale Palumbo, Nadia Rita Panico, Antonella Pansini, Roberta Pantieri, Paolo Paolello, Matteo Pardini, Lucilla Parnetti, Emma Parrotta, Michela Passamonte, Agostino Pastore, Ebe Pastorello, Luca Pelini, Morena Pellati, Mario Pellegrino, Giuseppe Pelliccioni, Maria Giovanna Pennisi, Michele Perini, Daniele Perotta, Diego Persico, Virginia Petrella, Fabia Petri, Maristella Piccininni, Laura Pierguidi, Alessio Pietrella, Alberto Pilotto, Patrizia Pinto, Alessandro Pirani, Vincenzo Pizza, Domenico Plantone, Massimiliano Plastino, Patrizia Poddighe, Simone Pomati, Angela Pompilio, Marialuisa Pontecorvo, Alessandro Prelle, Giorgio Previderè, Ennio Pucci, Gianfranco Puoti, Valeria Putzu, Annaflavia Rabasca, Massimo Raffaele, Innocenzo Rainero, Claudia Rais, Michele Rana, Alberto Ranzenigo, Giovanni Rea, Enrico Righetti, Giuseppe Rinaldi, Augusto Rini, Maria Rosaria Rizzo, Massimo Rizzo, Paola Rocca, Laura Roffredo, Daniela Roglia, Franco Romagnoni, Carlo Romano, Annalisa Romasco, Leonardo Romeo, Stefano Ronzoni, Chiara Emilia Rosci, Mara Rosso, Renzo Rozzini, Eleonora Ruberto, Stefania Ruberto, Gregorio Rungger, Giovanni Ruotolo, Francesco Russo, Giuseppe Russo, Roncacci Sabina, Simona Sacco, Giorgio Sacilotto, Giuseppe Salemi, Paolo Salotti, Elena Salvatore, Luisa Sambati, Giuseppe Sanges, Francesco Santamaria, Michele Ignazio Santilli, Mariangela Santoro, Riccardo Saponara, Monica Scarmagnan, Fabrizio Scataglini, Loredana Seccia, Vladimir Selmo, Stefano Sensi, Luigi Sicurella, Antonello Silvestri, Massimo Simone, Intissar Sleiman, Paolo Solla, Anna Sarah Sperber, Laura Spinelli, Franz Spoegler, Patrizia Sucapane, Domenico Suraci, Benedetta Tagliabue, Stefania Tagliente, Elena Tamietti, Gianluca Tedeschi, Antonio Tetto, Alessandro Tiezzi, Pietro Tiraboschi, Gloria Tognoni, Carmine Tomasetti, Francesco Torchia, Giuseppe Toriello, Giovanna Trevisi, Gabriele Tripi, Giuseppe Trombetta, Alessandro Tulliani, Rita Antonella Vaccina, Luca Valentinis, Gina Varricchio, Antonella Vasquez Giuliano, Filomena Vella, Federico Verde, Chiara Verlato, Giuliana Vezzadini, Simone Vidale, Assunta Vignoli, Daniele Villani, Alfredo Vitelli, Luigina Volpentesta, Gino Volpi, Domenico Vozza, Patrizia Wanderlingh, Christian Wenter, Davide Zaccherini, Massimo Zanardo, Giampietro Zanette, Michela Zanetti, Orazio Zanetti, Carla Zanferrari, Marta Zuffi, Vincenzo Zupo

**Affiliations:** 1 Italian National Institute of Health FONDEM Study Group, Rome, Italy; 2 Scientific Cultural Workshops, Cognitive Therapy Center (CTC), Como, Italy; 3 National Center for Disease Prevention and Health Promotion, Italian National Institute of Health, Rome, Italy; 4 Laboratory of Applied Psychology and Intervention, Department of Human and Social Sciences, University of Salento, Lecce, Italy; 5 Cooperativa Sociale TAM Onlus, Naples, Italy; 6 Department of Human Neuroscience, Sapienza University, Rome, Italy; 7 Center for Cognitive and Language Rehabilitation “Sinapsy”, Rome, Italy; 8 National Center for Drug Research and Evaluation, Italian National Institute of Health, Rome, Italy; 9 Department of Neurorehabilitation Sciences, Casa di Cura Igea, Milan, Italy

**Keywords:** Neuropsychological assessment, Dementia, Centers for Cognitive Disorders and Dementias, Public health, Survey, Minimum core test, National dementia plan, Memory clinic

## Abstract

**Background:**

The Italian Fund for Alzheimer’s and other dementias approved in 2020 enabled the conducting of a survey in the Italian Centers for Cognitive Disorders and Dementias (CCDDs) to analyse the organization, the administrative features and the professionals’ characteristics.

**Aims:**

To investigate the current use of neuropsychological (NP) tests in Italian CCDDs and the association between the use of a basic set of tests for neuropsychological assessment (NPA) and organizational/structural characteristics of CCDDs.

**Methods:**

A survey was conducted with an online questionnaire in all CCDDs between July 2022 and February 2023. To verify the use of a comprehensive NPA in the diagnosis of cognitive disorders and dementia, we identified a minimum core test (MCT).

**Results:**

The CCDDs using a Minimum Core Test (MCT) significantly increased from 45.7% in 2015 to the current 57.1%. Territorial CCDDs using MCT significantly increased from 24.9% in 2015 to 37% in 2022 (p = 0.004). As multivariable results, the presence of psychologist/neuropsychologist in the staff and the University-based/IRCCS CCDDs increased the probability of using MCT (OR = 9.2; 95% CI 5.6–15.0; p < 0.001 and OR = 5.4; 95% CI 1.9–15.9; p = 0.002, respectively), while CCDDs in Southern Italy-Islands showed a lower probability than those in the North (OR = 0.4; 95% CI 0.2–0.7; p = 0.001).

**Discussion:**

Almost half of CCDDs (43%) do not use MCT in their clinical practice. The presence of the psychologist/neuropsychologist on the staff has a key role in the adoption of MCT and regional differences have increased over the past years. NPA is crucial in the diagnostic process and in characterizing risk profiles in order to implement targeted interventions for risk reduction.

**Conclusions:**

Our results could help to identify good practices aimed at improving dementia diagnosis. An intervention by health policymakers is urgently needed with the aim of improving diagnostic appropriateness and overcoming regional differences.

**Supplementary Information:**

The online version contains supplementary material available at 10.1007/s40520-024-02869-6.

## Introduction

Dementia is worldwide recognised as a priority emergency in health- and social care. According to Alzheimer’s Disease International’s 2022 World Alzheimer Report, the global number of individuals living with dementia, which was estimated to be 55 million in 2019, is expected to increase to 139 million by 2050 [1]. Today, some countries around the world have developed their own national dementia strategies, which share the values of community awareness, personal and social responsibility, reliance on early diagnosis, and improved quality of care and life [2]. As noticed by the World Alzheimer Report, national dementia plans need to become a policy priority in order to ensure treatment, care and support for people with dementia [1]. Furthermore, the detection and timely diagnosis of Alzheimer’s disease (AD) and other neurodegenerative disorders are crucial for access to new therapies [3].

Neuropsychological assessment (NPA) plays a central role in the clinical evaluation of dementia risk (i.e., mild cognitive impairment or subtle cognitive changes not fulfilling the criteria for dementia), or dementia (i.e., dementia diagnosis according to a standard definition such as cognitive impairment impacting on social function and activities of daily living), and it is designed to determine the presence of cognitive and behavioral decline, the degree to which this decline interferes with functional daily activities but also identifying specific neurodegenerative diseases [4, 5].

The goal of the NPA is to identify whether a single cognitive domain is involved or whether the patient has multiple cognitive and behavioral deficits, reflecting the areas of the brain influenced by disease [6]. Brief measures for cognitive screening are currently widely used for a first-level examination in clinical practice [5]. The Mini Mental State Examination (MMSE) is a reliable and sensitive test for global cognitive function, although it has well-known limitations in distinguishing cognitively healthy individuals from subjects with mild dementia [7]. The Montreal Cognitive Assessment (MoCA) is another brief screening test with the advantage of also investigating executive functions and having good sensitivity for mild cognitive impairment and AD [8]. The Mini-Cog is a screening test consisting of a three-word recall task and the clock drawing test [9]. It is brief and easy to administer, but with limited information currently available on its diagnostic test due to the poor quality of the studies [10].

Screening tests should always be followed by a second-level assessment, which allows a detailed description of the patient's cognitive profile [11, 12]. Thus, a comprehensive NPA should include multiple cognitive domains, such as learning and memory, complex attention, executive functions, language, perceptual–motor function, and social cognition [13, 14].

However, there is a wide heterogeneity in cognitive tests and batteries used in clinical and research settings both among European countries and the different Italian regions [5, 15]. Some attempts were made in Europe to harmonize the use of neuropsychological (NP) tests across countries [4, 5, 16], highlighting how far we are from uniform our approach to cognitive assessment, due to out-of-date norms, the presence of linguistic minorities, not-comprehensive cognitive testing, differences in procedures of test administering and tests adopted among different countries or regions. Different tools are currently used to assess abilities such as memory, language and executive functions. However, the main issues raised in these surveys were about the psychometric solidity of the tools used. They emphasized the importance of being aware of some psychometric properties that NP tests should ideally fulfil such as content and construct validity [5].

The diagnosis and care of people with dementia in Italy is entrusted to Centers for Cognitive Disorders and Dementias (CCDDs). Previously established as Alzheimer’s Evaluation Units (UVA) and introduced with the Cronos project [17], the purposes of CCDDs were formulated by the Italian Dementia National Plan in 2014 [18]. The CCDDs are public services fully covered by the national health care system, involved in the timely recognition and diagnosis of cognitive disorders, responsible for prescribing drugs for behavioral and psychological symptoms of dementia (i.e., antipsychotics and antidepressants) and specific pharmacological treatments for AD (i.e., donepezil, rivastigmine, galantamine and memantine) [19], and focusing on the support of patients and families throughout the pathways of care [18].

In Italy, two surveys were conducted in 2002 and 2015 [20–22], carried out by the Italian National Institute of Health upon indication of the Ministry of Health with the purpose of investigating the activities conducted in the UVA [20] or in CCDDs [21, 22] and highlighting their central role in the diagnosis and treatment of people with dementia. They also investigated the type of tests used for the NPA. In 2002 the survey identified the main NPA instruments adopted by only 50% of the Italian facilities [20].

In 2015, the survey identified a minimum core test (MCT) to verify the use of a comprehensive NPA in the diagnosis of cognitive impairment and dementia and collected information on the presence or absence of a neuropsychologist or psychologist on the CCDD staff [21]. The MCT was described as an essential set of tests designed to assess key cognitive functions [21]. Results showed that only in 45.7% of the included CCDDs a comprehensive clinical evaluation was provided with large differences in the three macro-areas of the country [21, 22]. Thus, the reliability of dementia diagnosis varied significantly across different regions of the country.

Following the line of research already conducted in 2015, the present study aims to investigate how the situation has changed in Italy during the years in the third survey conducted in 2022, regarding the use of NP tests or MCT for the diagnosis of cognitive disorders and dementia in Italian CCDD’s, the presence of a psychologist or neuropsychologist in the staff, geographical macro-areas distribution and type of CCDD.

## Methods

The methodology of the study was described elsewhere [23]. Briefly, the survey was launched in July 2022 and closed in February 2023. A detailed list of CCDDs was provided by representatives of the Regions and Autonomous Provinces. The CCDDs were invited to participate in the study using a cover letter sent by e-mail.

The survey questionnaire consisted of two sections: a profile section and a data collection form [23]. In particular, the questionnaire was also used to gather information on the type of NPA tools and the clinical scales, tests and batteries used in CCDDs for the assessment and diagnosis of dementias. Information on the presence of at least one psychologist or neuropsychologist in the staff, geographic macro-areas [defined according to the categorization of the Italian National Statistics Institute (ISTAT), i.e., Northern Italy, Central Italy or Southern Italy and Islands], the type of CCDD [i.e., territorial services, hospital-based or University-based/IRCCS (Istituti di Ricovero e Cura a Carattere Scientifico)], and the use of a comprehensive NPA were included.

The questionnaire was approved by all representatives of the Permanent Table of the National Dementia Plan*.* Data were collected through a designed online platform, and exported for statistical analyses.

### Minimum core test

NP tests were classified according to their cognitive and functional domains. Based on compendia of cognitive testing and the recommendations from the Italian Neuropsychological Society [24] and the DSM-5 [13], NP tests were defined as follows: (1) learning and memory, language, perceptual-motor function, complex attention, executive functions and reasoning; (2) composite batteries for global assessment; (3) screening tests and (4) praxis. All tests were validated for the Italian population (Table 1).

**Table 1:** Overview of the use of neuropsychological tests in CCDDs in the three surveys

Domains and neuropsychological tests	2002 [20] Survey, %	2015 [21] Survey, %	2022 [23] Survey, %
	N = 392	N = 501	N = 450
*Learning and memory*			
Rey’s 15 words test [39]	11.0	65	70.7
Digit span [40, 41]	4.8	52.8	58.4
Corsi Block Test [40, 41]	12.0	46.4	45.8
Babcock short tale [42]	11.2	69.6	66.0
ROCF^a^ recall [42–44]	–	52.2	62.0
FCSRT^b^ [45, 46]	–	–	32.7
*Language*			
Semantic verbal fluency test [47, 48]	15.3	61.2	62.9
Token test [49, 50]	13.3	–	47.1
Boston Naming Test [51, 52]	–	–	23.6
AAT^c^ [53, 54]	1.5	17.8	20.0
Visual naming [55, 56]	–	17.0	15.6
*Perceptual–motor function*			
Drawings copy [39]	–	52.4	47.1
ROCF^a^ copy [42–44]	5.6	55.4	64.9
CDT^d^ [57, 58]	9.7	83.6	87.8
*Complex attention*			
Attentional matrices [49]	18.4	54.4	57.8
TMT^e^ A [59–61]	1.3	51.6	61.1
Line cancellation test [62]	–	–	16.9
Stroop test [63, 64]	2.3	33.2	45.1
*Executive functions and reasoning*			
FAS^f^ [39, 47]	17.1	61.8	68.2
CPM47^g^ [39, 65]	1.3	33.2	35.6
TMT^e^ B [59–61]	1.3	51.6	61.1
ToL^h^ test [66, 67]	–	–	25.8
RME^i^ test [68, 69]	–	–	10.0
SPM38^j^ [70, 71]	6.1	41.4	43.8
MCST^k^ [72, 73]	0.3	24.2	27.1
*Social cognition*			
Story-based empathy task [74]	–	–	8.4
*Composite batteries*			
MDB^l^ [39]	–	22.6	20.0
ACE-R^m^ [75, 76]	–	–	20.9
Benton Neuropsychological battery [77]	–	–	16.2
Short Neuropsychological Examination [78, 79]	–	–	34.7
FAB^n^ [80, 81]	–	–	69.8
ADAS-cog^o^ [82, 83]	24.0	2.4	36.4
MODA^p^ [84]	23.5	29.8	34.0
*Screening tests*			
MMSE^q^ [85, 86]	97.8	93.2	98.9
MoCA^r^ [8, 87, 88]	–	6.6	64
Mini-cog [9, 89]	–	–	18.2
*Praxis*			
Ideomotor apraxia [49]	–	–	39.1
Orofacial apraxia test [49]	–	–	32.7

^a^Rey-Osterrieth complex figure

^b^Free and Cued Selective Reminding Test

^c^Aachen Aphasie Test

^d^Clock drawing test

^e^Trail Making Test

^f^Phonemic verbal fluency test

^g^Raven's Progressive matrices color form

^h^Tower of London

^i^Reading the mind in the eyes

^j^Raven's Progressive matrices

^k^Modified card sorting test

^l^Mental Deterioration Battery

^m^Addenbroke's Cognitive Examination Revised

^n^Frontal Assessment Battery

^o^Alzheimer's disease assessment scale

^p^Milan overall dementia assessment

^q^Mini Mental State Examination

^r^Montreal Cognitive Assessment

In line with the previous survey [21], to verify the use of a comprehensive NPA in the diagnosis of cognitive disorders and dementia, we identified a minimum core test (MCT)—that is a set of at least one second-level task for each cognitive domain: both verbal and visual episodic memory, language, perceptual–motor function, complex attention and executive functions and reasoning. It should be mentioned, that MCT should always be preceded by a first-level screening.

According to previous studies [25, 26] and the recommendations from the Italian Neuropsychological Society [24], such an MCT would allow the detection of subtle cognitive impairments and different patterns of dementia based on the clinical criteria for the diagnosis of MCI or dementia [11, 12].

### Statistical analysis

The proportion of CCDDs using an MCT for the diagnosis of dementia was summarized by absolute frequencies and percentages and the Chi-square test was used for the comparison between the previous [21] and the current survey. A comparison of the distribution of facilities reporting the use of MCT according to (i) geographical macro-areas*,* (ii) presence of psychologists in the staff and (iii) type of CCDDs was performed using Chi-square test.

Logistic regression analysis was used to investigate the association between the use of an MCT and the three dependent variables above indicated. ORs and their 95% CIs were estimated by the model. P values lower than 0.05 were considered statistically significant. All statistical analyses were carried out using the Statistical Package STATA, v17.0, College Station, Texas, USA.

## Results

The survey was completed by 450 (84%) over 534 CCDDs (91% in the Northern Italy, 78% in the Central Italy, 81% in the Southern Italy and Islands) as described elsewhere [23].

### Neuropsychological assessment in the Italian Centers for Cognitive Disorders and Dementias

Table 1 reports data on the current use of NP tests in 450 Italian CCDDs and those from the previous studies conducted in 2002 (196 UVA over 392) and 2015 (501 CCDDs over 536) [20, 21]. Particularly, among participants CCDDs in 2022 the Rey’s 15 words test emerged as the most frequently used memory test, followed by the Babcock Short Tale and Rey-Osterrieth complex figure (ROCF) recall (see Table 1). In the language domain, the Semantic verbal fluency test was the prevailing choice (see Table 1). The Clock Drawing Test was the most commonly employed for assessing constructional abilities (see Table 1). Within the executive functions domain, the Phonemic verbal fluency test (FAS) was the predominantly used assessment tool (see Table 1). The MMSE and the MoCA were the prevalent screening tests utilized (see Table 1). These findings were very similar to the data collected in the 2015 survey only for the MMSE, while there was a large increase in the use of MoCA (from 6.6% in 2015 to 64% in 2022). However, in the complex attention domain, the Trail Making Test A (TMT A) resulted as the prevalent test, whereas the previous survey reported a prevalent use of Attentional Matrices and in the 2002 survey TMT A was marginally used (see Table 1). Finally, some neuropsychological (NP) tests appeared for the first time among the utilized tests in the 2022 survey, i.e., the Token Test, the Free and Cued Selective Reminding Test (FCSRT), the Boston naming test, the Line cancellation test, the Tower of London (ToL), the Reading the Mind in the Eyes (RME) test, the Story-based Empathy task, the ACE-R, the Benton battery, the Short Neuropsychological Examination, and the Mini-cog (see Table 1).

The aforementioned tests for each domain have been confirmed to be the most commonly used even when examining the detailed data for Italian geographical macro-areas (see Supplementary Table). However, the proportion of facilities administering NP tests varies significantly between Northern, Central, and Southern Italy. In particular, all NP tests assessing learning and memory, language, perceptual–motor function, complex attention and executive functions showed significant differences, with Northern Italy characterized by a more frequent use which decreased in the Central and further in the Southern Italy and Islands (p < 0.001 for all tests considered except Drawing copy test.

Among the composite batteries, the Frontal Assessment Battery (FAB) is the most used in all the CCDDs with a significantly higher adoption among those located in the Northern regions of Italy (85.6%) compared to Central (59.8%) and Southern Italy and Islands (55.4%, p < 0.001). Similarly, a more frequent use was found in the North macro-area for the short batteries: ACE-R (p = 0.001), Mental Deterioration Battery (MBD; p = 0.011), Short Neuropsychological Examination (p < 0.001), and for the screening test MoCA (p < 0.001).

The screening tests MMSE and Mini-cog were administered uniformly across all macro-areas (p = 0.976 and p = 0.541, respectively) (see Supplementary Table).

### Characterisation of CCDDs using MCT

A total of 257 CCDDs (57.1%) out of 450 used an MCT during their clinical practice (Table 2). This proportion marked a significant increase compared to the 2015 survey data, which reported 45.7% of facilities utilizing MCT (p < 0.001). Compared to the previous survey, there were no significant differences in the distribution by geographical location and the proportion of CCDDs with at least one psychologist or neuropsychologist. However, a notable change was observed in the type of facilities adopting MCT: the proportion of territorial CCDDs using MCT increased significantly from 24.9 to 37% (p = 0.004), between the previous and the current survey, whereas hospital-based facilities significantly decreased from 60.3 to 48.6% (p = 0.01) (Table 2).

**Table 2:** Comparisons between two surveys conducted in 2015 and 2022: CCDDs using a MCT according to geographical macro-areas, presence of at least one psychologist or neuropsychologist in the staff and type of CCDD

	2015	2022	p-value
CCDDs using MCT	229/501 (45.7%)	257/450 (57.1%)	0.001
	*N = 229*	*N = 257*	
*Psychologist in the CCDD staff*			
At least one	187 (81.7%)	223 (86.8%)	0.122
No psychologist or neuropsychologist	42 (18.3%)	34 (13.2%)	0.122
*Geographical macro-area of CCDD*			
Northern Italy	127 (55.5%)	149 (58.0%)	0.579
Central Italy	44 (19.2%)	43 (16.7%)	0.473
Southern Italy and Islands	58 (25.3%)	65 (25.3%)	1.000
*Type of CCDD*			
Territorial services	57 (24.9%)	95 (37.0%)	0.004
Hospital-based	138 (60.3%)	125 (48.6%)	0.010
University-based/IRCCS	34 (14.8%)	37 (14.4%)	0.901

*MCT*  minimum core test, *CCDD*  Centers for Cognitive Disorders and Dementias, *IRCCS*  Istituti di Ricovero e Cura a Carattere Scientifico

### Logistic regression results

As reported in Table 3, the probability of using an MCT is significantly higher in the CCDDs including at least one psychologist or neuropsychologist in their team, this association remained significant also after adjustment for the other variables (OR 9.2; CI95% 5.6–15.0; p < 0.001). Hospital-based facilities and university-based/IRCCS CCDDs showed a higher probability of using MCT compared to territorial, however only the latter remained significantly associated in the multivariable model (OR 5.4; CI 95% 1.9–15.9; p = 0.002). In contrast, the probability of using an MCT was more than halved in CCDDs located in Central and Southern Italy and the Islands in comparison with facilities located in the Northern regions. After the multivariable adjustment, the probability of using an MCT was 60% lower when comparing CCDDs located in Southern Italy and the Islands to those in the North (OR 0.4; CI 95% 0.2–0.7; p = 0.001). This finding showed worsening with respect to the 2015 survey (OR 0.56; CI 95% 0.35–0.89; p = 0.014).

**Table 3:** Descriptive analysis and logistic regression assessing the association between the use of MCT and CCDDs’ characteristics in the survey conducted in 2022

	N (%)	Univariable analysis			Multivariable analysis		
		OR	95%CI	p-value	AOR	95%CI	p-value
*At least one psychologist/ neuropsychologist in the staff*	291 (64.7%)	12.1	7.6–19.2	< 0.001	9.2	5.6–15.0	< 0.001
*Type of CCDD*							
Territorial	200 (44.4%)	1.0			1.0		
Hospital-based	208 (46.2%)	1.7	1.1–2.5	0.011	1.4	0.8–2.2	0.203
University-based/IRCCS	42 (9.3%)	8.2	3.1–21.7	< 0.001	5.4	1.9–15.9	0.002
*Geographic macro-area*							
Northern Italy	202 (44.9%)	1.0			1.0		
Central Italy	82 (18.2%)	0.4	0.2–0.7	0.001	0.7	0.3–1.3	0.226
Southern Italy and Islands	166 (36.9%)	0.2	0.1–0.4	< 0.001	0.4	0.2–0.7	0.001

*OR*  odds ratio, *AOR*  adjusted odds ratio, *95% CI*  95% confidence interval for the estimated odds ratio, *MCT*  minimum core test, *CCDD*  Centers for Cognitive Disorders and Dementias; *IRCCS*  Istituti di Ricovero e Cura a Carattere Scientifico

## Discussion

The present survey provides an up-to-date overview of the use of NPA in Italian CCDDs and the characteristics of CCDDs adopting the MCT for NPA. A comprehensive cognitive assessment is crucial in the diagnostic process of cognitive impairment and dementia. However, in the European scenario, data on tools used in health services for the assessment of dementias are scarce and studies included a very limited number of centers [4, 27]. As previously noticed [23], a remarkable response rate of 84% was obtained thanks to the close collaboration between the Italian National Institute of Health, the regional institutional representatives, and health professionals in charge of CCDDs. Then, to our knowledge, there are no studies similar to ours on the use of NP tests in the current clinical practice of memory clinics in other countries. This prevents us from making comparisons with other healthcare settings and therefore having a more general picture of the phenomenon.

Firstly, important finding, our results confirm the trend, already highlighted in the 2015 survey [21], to use more key tools for the diagnosis of MCI and dementia, such as tests for episodic memory, language, complex attention, perceptual–motor function, executive functions and social cognition, in line with the Diagnostic and Statistical Manual of Mental Disorders, Fifth Edition (DSM-5) guidelines [13], and with the clinical criteria for the diagnosis of MCI or dementia [11, 12].

Moreover, the publication of the normative data in the Italian population encouraged the adoption of some internationally well-known NP tests (i.e., FCSRT, Boston Naming test, Tower of London test, RME test and Story-based Empathy task). In particular, among memory tests, the FCSRT aims to the episodic memory assessment in elderly people under conditions that control the encoding effectiveness. Two episodic memory tests incremented their utilization: the Rey’s 15 words test, a well-recognized measure of a person's ability to encode, store and recover verbal information in different stages of immediate memory, and the ROCF recall, i.e., the delayed recall trial of the ROCF test measuring the visual memory. These results highlighted a growing awareness of the importance of deeply evaluating episodic memory impairment, in line with the current clinical and research criteria for the diagnosis of Alzheimer’s dementia [28, 29]. In the language domain, compared to the previous surveys, the utilization of the Token test, assessing auditory comprehension, was increased not only because its utility in defining cognitive profiles of subjects with language disorders [30], but also in assessing the progression of cognitive impairment in AD [13]. In the perceptual–motor function domain, the ROCF copy was adopted by a higher number of CCDDs than in the past. The ROCF copy, measuring the perceptual–motor function and visuospatial construction ability by means of the copy trial, is effective in the differential diagnosis of dementias, even in the earliest stages [31]. The Stroop test and TMT-A in the complex attention domain and the TMT-B in the executive functions domain incremented their utilization. They assess a wide range of active cognitive processes including problem-solving, planning, sustained attention, inhibition, multitasking, cognitive flexibility, and the ability to cope with novelty: we could hypothesize that the increased interest in assessing this cognitive area is linked to the recent studies confirming the presence of early impairment in AD subjects [32]. Among the composite batteries for global cognition, ADAS-cog was adopted by a higher number of CCDDs than in the previous surveys, probably due to its ability to capture cognitive decline in follow-up assessment, although this tool was widespread in clinical trials [33]. Furthermore, the ACE-R, and the FAB started to be used by many services in 2022 probably thanks to the recent updating of normative data in the Italian population [34].

In 2022 we surveyed utilization in the NPA of CCDDs of two tests of social cognition because of the growing literature on its impairment in neurodegenerative diseases [35, 36]. The prevalence of the RME test and the Story-based Empathy task utilization is still low despite the fact that social cognition is recognized as a diagnostic criterion of neurocognitive disorders [13].

Our results on cognitive domains assessed in memory clinics confirm the accordance with previous data from other European countries [4, 21]. Moreover, our results are in line with results from the consensus conference on NPA for neurocognitive disorders across European countries to enhance the NP battery’s sensitivity to the typical AD, atypical AD, and behavioral variant of frontotemporal dementia [37]. However, there are many disparities in the use of NP tests between the different Italian macro-areas, with a lower adoption of NP tests in Southern Italy and the Islands compared to the Northern regions and Central Italy (see Supplementary Table). This reflects wide differences in the allocation of healthcare resources, in terms of geographical distribution of the centers and number of dedicated professionals.

The second finding is that the overall use of the MCT is significantly higher than in the past (going from 45% in 2015 to the current 57%), possibly ascribed to the scientific and cultural process of harmonization of cognitive assessment for detection of cognitive impairment and dementia. These encouraging results indicate overall growth throughout our country because of the higher use of the MCT in CCDDs of Northern Italy, and the increase in territorial services. However, there are still too many inequalities across regions about health services and NP instruments currently adopted for dementia diagnosis. Several factors may explain the lower likelihood of receiving an NPA with an MCT in the South and Islands. One reason could be the increase in the number of patients in charge. As previously reported (23), we found that the total number of patients yearly seen in a CCDD increased by 10% between 2014 and 2019, with a notable increase in the South. Another possible factor is the lower availability of an integrated care pathway in the South [23].

Our results could help to identify good practices aimed at improving early dementia diagnosis and differential diagnosis in order to improve dementia care and minimize patient and family burdens.

The third important result of the present survey is the improvement over 2015 in the presence of at least one psychologist or neuropsychologist in the CCDDs’ team in association with a higher probability of using an MCT in the CCDDs (going from OR = 4.55 in 2015 to the current OR = 9.2). As previously reported [21], in Italy professional psychologists’ expertise includes the specific requirements for neuropsychological examiners, already defined in 1985 by the American Psychological Association. Currently, as we are far from having comprehensive NPA as part of the health offered by Italian memory clinics, public efforts toward increasing professional figures such as psychologists are needed. Besides behavioral and functional assessment, the comprehensive NPA is crucial in characterizing the cognitive profiles of individuals at risk (i.e. with mild cognitive impairment or subtle cognitive changes) or individuals with dementia (i.e. meeting the criteria for dementia), quantifying cognitive impairment, detecting relevant change over time, and planning targeted interventions [5, 14, 37]. Furthermore, early identification of risk factors in fully evaluated individuals with appropriate NP testing would allow the implementation of targeted interventions to reduce the risk of dementia, an action recently supported by the European Task Force for Brain Health Services [38]. The advent of new drug therapies for dementia will require a high selection of eligible patients with a correct diagnosis [38]. Thus, it will be important to act as soon as possible to improve the NPA offered by CCDDs to enable patients to receive therapies. Finally, from a public health point of view, an intervention by national and local health authorities and scientific societies involved in the topic of dementia is urgently needed with the aim of improving diagnostic appropriateness considering that 43% of CCDDs do not declare an MCT and the large differences regional in the use of NP tests. Policymakers’ interventions are more urgent because our findings highlighted a worsening over the past years in the probability of using an MCT among CCDDs located in the Southern regions and the Islands compared to Northern Italy.

The main strength of this survey is the inclusion of structures based on the whole national territory. Our data showed remarkable reliability thanks to the high participation rate of CCDDs in the three surveys (2002 [20], 2015 [21], 2022 [23]). This study can be of support in understanding the functioning of Italian CCDDs and the type of NP tools used in clinical practice to assess people with cognitive complaints. This is an extremely relevant issue, considering that potentially disease-modifying treatments are currently under development, that will require more sensitive NP measures for the early identification of cognitive disorders and dementia. The main limitation of this survey is the use of self-administered questionnaires, thus potentially overestimating the actual scenario.

## Conclusions

Our results on NPA could help to identify good practices aimed at improving early dementia diagnosis and differential diagnosis. 43% of CCDDs do not declare an MCT and there are large disparities between Northern and Southern Italy and the Islands. The adoption of MCT as part of a high-quality health offer is linked to the presence of the psychologist or neuropsychologist in the CCDD’s staff. Such professionals have key importance because the role of NPA is crucial in the diagnostic process and in characterizing relative risk profiles in order to implement targeted interventions for dementia risk reduction. An intervention by health policymakers is urgently needed with the aim of improving diagnostic appropriateness and overcoming regional differences in the use of NP tests.

## Supplementary Information

Below is the link to the electronic supplementary material.

Supplementary Material 1.

## Data Availability

The data supporting the findings of this study are available on request from the corresponding author.
