# Supplementary material for: Neuropsychological tests at the Italian Centers for Cognitive Disorders and Dementias: results from a survey on 450 specialized services
Source: Aging Clin Exp Res. 2024 Dec 20;37(1):1. doi: 10.1007/s40520-024-02869-6 (PMC11662035; doi:10.1007/s40520-024-02869-6)
Supplement: Supplementary file 1 — Supplementary Material 1. [file 40520_2024_2869_MOESM1_ESM.docx]

Supplementary table. Use of neuropsychological tests in CCDDs according to geographical macro-areas (survey 2022).

| **Domains and neuropsychological tests** | **Total**  **N=450** | **Northern Italy**  **N=202** | **Central Italy**  **N=82** | **Southern Italy and Islands**  **N=166** | **p value** |
| --- | --- | --- | --- | --- | --- |
| ***Learning and Memory*** |  |  |  |  |  |
| Corsi block test | 45.8% | 58.4% | 43.9% | 31.3% | <0.001 |
| Digit Span | 58.4% | 73.8% | 57.3% | 40.4% | <0.001 |
| Babcock short tale | 66.0% | 77.7% | 64.6% | 52.4% | <0.001 |
| Free and Cued Selective Reminding Test (FCSRT) | 32.7% | 49.5% | 34.1% | 11.4% | <0.001 |
| Rey’s Auditory Verbal Learning Test (RAVLT) | 70.7% | 81.7% | 69.5% | 57.8% | <0.001 |
| Rey-Osterrieth complex figure (ROCF recall) | 62.0% | 77.7% | 56.1% | 45.8% | <0.001 |
| ***Language*** |  |  |  |  |  |
| Aachen Aphasie Test (AAT) | 20.0% | 28.7% | 13.4% | 12.7% | <0.001 |
| Boston Naming Test | 23.6% | 32.7% | 29.3% | 9.6% | <0.001 |
| Semantic verbal fluency test | 62.9% | 79.2% | 59.8% | 44.6% | <0.001 |
| Visual naming | 16.7% | 29.7% | 9.8% | 4.2% | <0.001 |
| Token test | 47.1% | 65.3% | 46.3% | 25.3% | <0.001 |
| ***Perceptual–motor function*** |  |  |  |  |  |
| Clock drawing test (CDT) | 87.8% | 96.0% | 86.6% | 78.3% | <0.001 |
| Drawings copy | 47.3% | 52.5% | 48.8% | 40.4% | 0.066 |
| Rey-Osterrieth complex figure (ROCF copy) | 64.9% | 82.2% | 57.3% | 47.6% | <0.001 |
| ***Complex attention*** |  |  |  |  |  |
| Trail Making Test (TMT A) | 61.1% | 78.7% | 59.8% | 40.4% | <0.001 |
| Attentional matrices | 57.8% | 71.8% | 53.7% | 42.8% | <0.001 |
| Line cancellation test | 16.9% | 26.2% | 15.9% | 6.0% | <0.001 |
| Stroop Test | 45.1% | 56.9% | 51.2% | 27.7% | <0.001 |
| ***Executive functions*** ***and Reasoning*** |  |  |  |  |  |
| Trail Making Test (TMT B) | 61.1% | 78.7% | 59.8% | 40.4% | <0.001 |
| Raven's Coloured Progressive matrices (CPM47) | 35.6% | 47.5% | 35.4% | 21.1% | <0.001 |
| Raven's Standard Progressive matrices (SPM38) | 43.8% | 57.4% | 42.7% | 27.7% | <0.001 |
| Phonemic verbal fluency test (FAS) | 68.2% | 85.6% | 64.6% | 48.8% | <0.001 |
| Modified card sorting test (MCST) | 27.1% | 34.7% | 31.7% | 15.7% | <0.001 |
| Tower of London test (ToL) | 25.8% | 36.6% | 25.6% | 12.7% | <0.001 |
| Reading the mind in the eyes (RME test) | 10.0% | 16.3% | 8.5% | 3.0% | <0.001 |
| ***Social cognition*** |  |  |  |  |  |
| Story-based Empathy task | 8.4% | 12.9% | 8.5% | 3.0% | <0.001 |
| ***Composite Batteries*** |  |  |  |  |  |
| Addenbroke's Cognitive Examination Revised (ACE-R) | 20.9% | 28.7% | 18.3% | 12.7% | 0.001 |
| Alzheimer's disease assessment scale (ADAS-cog) | 36.4% | 33.7% | 39.0% | 38.6% | 0.541 |
| Benton Neuropsychological battery | 16.2% | 20.3% | 14.6% | 12.0% | 0.093 |
| Frontal Assessment Battery (FAB) | 69.8% | 85.6% | 59.8% | 55.4% | <0.001 |
| Mental Deterioration Battery (MDB) | 20.0% | 24.8% | 23.2% | 12.7% | 0.011 |
| Milan overall dementia assessment (MODA) | 34.0% | 39.1% | 26.8% | 31.3% | 0.093 |
| Short Neuropsychological Examination | 34.7% | 49.0% | 24.4% | 22.3% | <0.001 |
| ***Screening tests*** |  |  |  |  |  |
| Montreal Cognitive Assessment (MoCA) | 64% | 78.2% | 56.1% | 50.6% | <0.001 |
| Mini Mental State Examination (MMSE) | 98.9% | 99.0% | 98.8% | 98.8% | 0.976 |
| Mini-cog | 18.2% | 22.8% | 18.3% | 12.7% | 0.044 |
| ***Praxis*** |  |  |  |  |  |
| Ideomotor apraxia | 39.1% | 50.0% | 48.8% | 21.1% | <0.001 |
| Orofacial apraxia | 32.7% | 40.1% | 43.9% | 18.1% | <0.001 |
